# Effects of intergenerational contact on social capital in community-dwelling adults aged 25–84 years: a non-randomized community-based intervention

**DOI:** 10.1186/s12889-022-14205-6

**Published:** 2022-09-24

**Authors:** Yuta Nemoto, Kumiko Nonaka, Masataka Kuraoka, Sachiko Murayama, Motoki Tanaka, Hiroko Matsunaga, Yoh Murayama, Hiroshi Murayama, Erika Kobayashi, Yoji Inaba, Shuichiro Watanabe, Kazushi Maruo, Yoshinori Fujiwara

**Affiliations:** 1 Research Team for Social Participation and Community Health, Tokyo Metropolitan Institute of Gerontology, 35-2 Sakae-cho, Itabashi, Tokyo, 173-0015 Japan; 2 Department of Preventive Medicine and Public Health, Tokyo Medical University, 6-1-1 Shinjuku, Shinjuku, Tokyo 160-8402 Japan; 3 College of Health and Welfare, J. F. Oberlin University, 3758, Tokiwamachi, Machida, Tokyo 194-0213 Japan; 4 Faculty of Medicine, University of Tsukuba, 1-1-1 Tennodai, Tsukuba, Ibaraki 305-8575 Japan

**Keywords:** Community-based intervention, Intergenerational contact, Urban area, Social trust, Norm of reciprocity, Social support

## Abstract

**Background:**

Accumulating social capital in urban areas is essential to improve community health. Previous studies suggested that intergenerational contact may be effective for enhancing social capital. However, no study has examined the effect of intergenerational contact on social capital through a population-based evaluation. This study aimed to investigate the effects of a community-based intervention to increase the frequency of intergenerational contact on social capital among adults aged 25–84 years.

**Methods:**

This study used a non-randomized controlled trial design to conduct a community-based intervention (from March 2016 to March 2019). The study area was Tama ward, Kawasaki city, Kanagawa, Japan. The area comprises five districts; one district was assigned as the intervention group and the other four districts as the control group. We provided the intervention to residents in the intervention group. The intervention comprised three phases: Phase 1 was the preparation term (organizing the project committee); Phase 2 was the implementation term (trained volunteer staff members, conducted the intergenerational greeting campaign, and held intergenerational contact events); and Phase 3 was the transition term (surrendering the lead role of the project to the city hall field workers). In the control group, field workers provided public health services as usual. We conducted mail surveys in September 2016 and November 2018 to assess the effects of the intervention on social capital during Phase 2. Eligible participants were randomly selected from community-dwelling adults aged 25–84 years according to age (10,620 control group individuals and 4479 intervention group individuals). We evaluated social trust, norm of reciprocity, and social support as outcome variables.

**Results:**

In total, 2518 participants completed both surveys and were analyzed (control group: 1727; intervention group: 791). We found that social trust (coefficient = 0.065; 95% confidence interval [CI]: 0.006, 0.125) and norm of reciprocity (coefficient = 0.084; 95% CI: 0.020, 0.149) positively changed in the intervention group compared with the control group.

**Conclusions:**

This community-based intervention may contribute to sustaining and improving social capital among community-dwelling adults.

**Trial registration**: UMIN000046769 (UMIN-CTR); first registered on January 28, 2022 (retrospectively registered).

**Supplementary Information:**

The online version contains supplementary material available at 10.1186/s12889-022-14205-6.

## Introduction

Many community-dwelling adults in Japan do not have social support resources. For example, among mothers of preschool children, 26.2% did not have opportunities to talk to someone about parenting issues, and 42.9% could not ask someone to look after their children for a short time [1]. Among older men who did not have children, 35.0% could not rely on someone when they needed help [2]. This social environment leads to social isolation and loneliness, and objectively and subjectively isolated adults have a higher risk for depression, coronary heart disease, and mortality [3–5].

Social capital, or social organization features that facilitate action and cooperation for mutual benefit [6], is essential for reducing social isolation and loneliness. Previous studies reported that individuals with higher-level social trust were less likely to be socially isolated than those with lower-level social trust [7]. However, social capital has decreased over the past several decades [8], and is often less accumulated in urban areas than in rural areas [9, 10]. Establishing social relationships and improving social capital in urban areas would be essential to prevent social isolation and enhance community health.

Although contact frequency and duration are determinants of the strength of social relationships [11], spontaneous interaction is not adequate to establish social relationships among community-dwelling adults. Among Japanese adults living in an urban area, 34.1% of young adults (aged 25–49 years) and 22.1% of older adults (aged 65–84 years) had no regular contact with neighbors; only 16.5% of young adults and 29.9% of older adults regularly had intra- and intergenerational communication [12]. Intergenerational programs, which promote contact between people from different age groups, may enhance social capital. Such programs typically involve scheduled activities (e.g., reading picture books) that are designed to bring older and younger generations together for the benefit of all participants [13]. Many previous studies reported that intergenerational programs improved older adults’ physical and cognitive function [13], reduced ageism [14, 15], and enhanced social capital (i.e., social support, norm of reciprocity, and social trust) [13, 16]. Therefore, interventions promoting intergenerational contact may reduce discrimination toward others based on age [13], promote trust in other generations and neighbors, and improve residents’ physical and mental health.

However, some aspects of the association between intergenerational contact and social capital remain unclear. First, no study has conducted a community-based intervention to examine the impact of intergenerational contact on social capital among the general population. Most intergenerational program studies enrolled a small number of participants and biased populations [15]. Although a prior cross-sectional study examined the association between the duration of intergenerational programs and social capital using a population-based evaluation [16], that study could not detect a causal effect. Second, few studies have examined whether intergenerational interaction between young and older adults was beneficial for improving social capital [17]. Intergenerational contact is important for children and older adults and for young and middle-aged adults [17]. However, most previous studies focused on the intergenerational relationship between kindergarten or school children and older people. Addressing this knowledge gap would contribute to developing a strategy to promote intergenerational communication to improve community-level social capital.

We conducted a community-based intervention to increase intergenerational contact, which was named the “Nakanoshima multi-generational relationship project.” This study aimed to examine the intervention effect on social capital among young to older adults through a population-based evaluation. We hypothesized that the intervention would enhance social trust, norm of reciprocity, and social support.

## Methods

### Study design, study setting, and allocation

This non-randomized controlled trial involved a community-based intervention (from March 2016 to March 2019) for community-dwelling adults aged 25–84 years. The study area was Tama ward, Kawasaki city, Kanagawa, Japan. This is an urban area with a population of 206,658 people in 2016 and an aging rate of 19.1%. The region is a typical commuter city in the western suburb of the Tokyo metropolitan area. The area comprises five districts. The median (range) population was 40,908 (23,000–73,608) people, among whom 7695 (3910–15,468) were aged ≥ 65 years.

In March 2016, the researchers and city hall field workers assigned one district (population: 23,000; aging rate: 20.4%) as the intervention group and the other four districts as the control group. In the intervention group, there were several housing complexes in which most residents were older adults in the north area, whereas many nuclear families lived in the south area. Therefore, the field workers believed intergenerational interactions in this area were low. Furthermore, the local community association leader was concerned about the lack of intergenerational relationships among residents and consented to the community-based intervention. Therefore, we allocated this district to the intervention group.

**Fig. 1:**
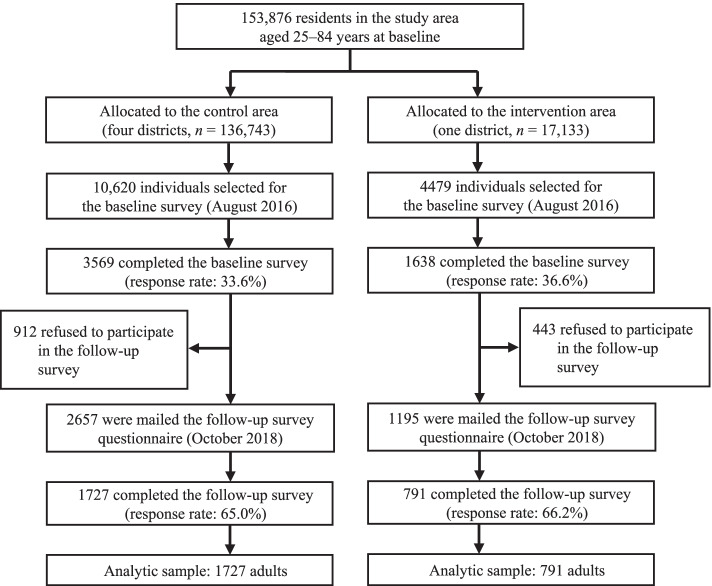
Flow chart of this study

We provided an intervention targeting young, middle-aged, and older adults in the intervention group from March 2016 to March 2019, and conducted a population-based evaluation to assess the intervention effect. Mail surveys were conducted in September 2016 (baseline) and November 2018 (follow-up). Eligible participants for the baseline survey were randomly selected from community-dwelling adults aged 25–84 years according to age. Since the response rate for younger adults (aged 25–49 years) was expected to be low, we oversampled that age group. We sampled 7549 younger adults, 3773 middle-aged adults (aged 50–64 years), and 3777 older adults (aged 65–84 years). In total, 15,099 adults (10,620 in the control group, and 4479 in the intervention group) were selected. Participants were asked to complete the self-administered questionnaire at baseline and to participate in the follow-up assessment. Those who refused to participate in the follow-up survey were excluded. Of 15,099 adults, 5207 individuals responded to the baseline survey. The response rate in the control group was 33.6%, and that in the intervention group was 36.6%. In total, 1355 individuals refused to participate in the follow-up survey (control group: 912; intervention group: 443), meaning 3852 individuals were asked to respond to the follow-up survey (control group: 2657; intervention group: 1195). Overall, 2518 participants completed both surveys and were included in the analyses (control group: 1727; intervention group: 791) (Fig. 1).

This study was conducted in accordance with the Declaration of Helsinki and approved by the Ethics Committee of the Tokyo Metropolitan Institute of Gerontology (protocol code 28KEN-1042; date of approval: 1 June 2016). The study was retrospectively registered in the UMIN Clinical Trials Registry (UMIN000046769).

### Intervention

The intervention period was from March 2016 to March 2019. The intervention was designed to be maintained after the study period. Our previous survey suggested that the project committee, which managed the intervention, would be required to maintain the intervention program for the long term [18]; therefore, we established the project committee and conducted the intervention to promote intergenerational contact.

The intervention period comprised three phases: Phase 1 was the preparation term (from March 2016 to August 2016); Phase 2 was the implementation term (from September 2016 to November 2018); Phase 3 was the transition term (from December 2018 to March 2019). As Phase 2 was the main part of the intervention, the changes in outcome variables during Phase 2 were examined as the intervention effects. We provided the following intervention to the residents in the intervention group. In the control group, city field workers provided public health services as usual.

### Phase 1: preparation term

In this term, we organized the project committee and held a monthly meeting to prepare the intervention.

### Project committee

The committee was organized to plan and manage the intervention to increase the frequency of intergenerational contact. The members comprised city hall field workers, neighborhood community association leaders, senior club leaders, volunteer group leaders, representatives of a nursing care home, and schoolteachers (nursery, elementary, and junior high school). Most invited members had a wealth of experience in managing intergenerational events such as annual local music festivals and welfare festivals. In addition, they knew each other through these prior experiences and had already developed cooperative relationships. Seventeen committee meetings were held. We held the meeting once a month from March 2016 to March 2017 and every two to three months from May 2017 to March 2019. The committee decided to conduct an intergenerational greeting campaign and intergenerational contact events. Before the meetings, the researchers and core committee members (i.e., city hall field workers and the senior club representative) discussed the framework of the intervention program. The committee members developed a concrete conducting plan at these meetings.

During the implementation term, the committee members participated in providing the intervention (e.g., committee members solicited their neighbors to be volunteer staff). In addition, they educated their neighbors and community members about the importance of intergenerational mutual help relationships. Moreover, they held a community event in February 2018 to introduce the purpose and contents of the “Nakanoshima multi-generational relationship project” and the project’s achievement.

### Phase 2: implementation term

In phase 2, we recruited and trained volunteer members, after which we conducted an intergenerational greeting campaign and intergenerational contact events.

### Volunteer staff members

We conducted training for the volunteer members, who had the role of planning and managing the intergenerational contact events. They completed seven classes to learn essential knowledge to promote intergenerational contact and build intergenerational relationships. These classes covered: explaining the purpose and contents of the “Nakanoshima multi-generational relationship project”; supporting approach for adults who were parenting children (two classes); supporting approach for older adults; how to promote intergenerational interaction (two classes); and group discussion.

In total, 18 individuals completed the course and became volunteer members. They met once a month to discuss plans to launch the intergenerational contact events, and held 63 events during the implementation term.

### Intergenerational greeting campaign

As contact frequency and duration strengthen social relationships [11], and greeting activity is a widespread activity that connects schools and communities in Japan [19], we performed a community-wide campaign to increase the frequency of intergenerational greeting. This aimed to raise awareness of the importance of greeting neighbors of different generations among residents and increase the frequency of intergenerational contact that occurred as part of people’s daily routine [17].

First, we developed a slogan and logo for this campaign, which emphasized the importance of intergenerational greeting (Supplementary Fig. 1). To attract people’s attention, we held a contest to select a slogan and logo, where elementary and junior high school students submitted their ideas. In the evaluation process, project committee members chose the best slogan and logo from the candidates, and the winners were awarded at a local event. The contest was introduced in the local newspaper. Next, we used the slogan and logo on printed goods, such as badges, stationery, banners, and uniforms (Supplementary Fig. 2). Brochures, badges, and stationery were distributed to elementary and junior high school students and their parents and to residents at existing local events. These events included the annual music festival, which took place over the last 10 years and was run by volunteer groups, and the annual welfare festival, which was held by the local social welfare association. Banners were displayed at school gates and the train station. Committee members also gave a lecture on intergenerational greeting at the local music festival (four times) and conducted greeting campaigns at school entrance ceremonies. Furthermore, we asked volunteer groups to wear the campaign uniform while watching over children returning home from school. Watching over school children is a typical volunteer activity among Japanese older adults, and this took place once a week in the study area.

### Intergenerational contact events

We held intergenerational contact events for young to older adults to meet, interact, and build trust and friendship with neighbors of other generations [19]. Previous studies [13, 14, 17] suggested that sharing recreational activities with different age groups effectively fostered intergenerational relationships. Therefore, the events included recreational activities (e.g., games, handcrafts, stretching, walking) and intergenerational communication.

The trained volunteer members launched the three intergenerational contact spaces and held events once or twice a month in each space. One of these spaces was the “Kamifuda café” in the northwest area of the intervention district, where events were held once a month; participants were served drinks and snacks, and enjoyed talking with other participants. Another space was “Pole de walk,” which was located in the northeast area and held twice a month; participants took a walk using Nordic walking poles and performed muscle strength training. The third space was the “Nakanoshima family café,” which was located in the south area and held twice a month. Participants in this event engaged in various recreational activities, such as handcrafts, stretching, yoga, seasonal events (e.g., Christmas party, Halloween party), and intergenerational communication. The total number of participants in these events was 794.

The events allowed participants to share the experience with their family, friends, and neighbors. Therefore, we hypothesized that the influence of the intergenerational contact events may expand beyond the actual number of participants.

### Phase 3: transition term

The researchers managed the project committee and trained volunteer members in Phases 1 and 2. In Phase 3, we surrendered these roles to the city hall field workers to continue the intervention program after the study period. We supported the field workers in organizing meetings and managing the volunteer members during this term.

### Measurements

#### Awareness of the community-based intervention

We assessed awareness of the intervention among the intervention group. Participants were asked: whether they knew the project title (“Nakanoshima multi-generational relationship project”), whether they knew the slogan and logo, and whether they had goods such as badges and stationery. In addition, participants reported whether they knew about and participated in intergenerational contact events, including Kamifuda café, Pole de walk, and Nakanosima family café. Possible answers were “I have participated,” “I have not participated, but I know the events,” and “I do not know the events.” Participants who knew the title/logo/slogan, had goods, or knew about the intergenerational contact events were classified as “individuals who perceived the intervention.”

#### Social capital

Although the concept of social capital has not been clearly determined, it has several dimensions, including social trust, norm of reciprocity, and social support. Each factor has a protective effect on health outcomes [20–22]. Therefore, we evaluated these indicators as the outcome variables.

Social trust and norm of reciprocity were assessed with one item each (i.e., “People in your neighborhood can be trusted” and “People in your neighborhood help each other”). Possible answers were: 1 = “agree,” 2 = “somewhat agree,” 3 = “neither agree nor disagree,” 4 = “somewhat disagree,” or 5 = “disagree.” We reversed the score to indicate that a higher score indicated better social trust and norm of reciprocity. The total score ranged from 1 to 5.

We assessed the frequencies of exchanging emotional and instrumental support as social support. Emotional social support was measured by how often the participants listened to others’ issues (support provided) or other people listened to the participants’ issues (support received). First, the participants reported the exchange frequency against each age group (20–49 years, 50–69 years, ≥ 70 years) by selecting an answer from: 1 = “often,” 2 = “sometimes,” 3 = “rarely,” and 4 = “not at all.” Then, we reversed the scores, summed the frequency, and calculated the average value of reciprocal social support (scores ranged from 3 to 12). Instrumental social support was measured by how often the participants helped to overcome someone’s problems (support provided) or other people helped overcome the participants’ issues (support received). Possible answers and score computation were the same as for emotional support.

#### Covariates

Covariates included sociodemographic variables and health status. Sociodemographic variables were sex, age, years of education (< 13 years, ≥ 13 years), household income (do not know, < 1 million yen, 1–2 million yen, 2–3 million yen, 3–5 million yen, 5–7 million yen, 7–10 million yen, ≥ 10 million yen), employment status (workers, non-workers), marital status (married, widowed/divorced/single), and living arrangement (living alone, living with someone). Health status included mental health and self-rated health (good, poor). Mental health was evaluated using the World Health Organization-Five Well-Being Index [23]. The measure is widely used for assessing subjective psychological well-being. The measurement comprises five items, and the score ranges from 0 to 25. We treated this score as a continuous variable.

#### Statistical analysis

We used descriptive statistics to summarize participants’ characteristics with means (standard deviation [SD]) for continuous variables and percentages for categorical variables. We examined group differences in baseline characteristics with independent t-tests for numerical variables and chi-square tests for categorical variables.

In this study, 26.0% of baseline survey responders refused to participate in the follow-up survey, and 2689 (51.6%) participants were lost to follow-up. We confirmed the difference in the characteristics between those lost to follow-up and those included in the primary analyses by conducting Poisson regression model with robust variance.

We performed analysis of covariance (ANCOVA) to assess the intervention effects on social capital. Although the outcomes were Likert scales, we used a parametric analysis because this method is robust with non-normal distributions [24]. The outcome variable of the models was the change in outcome variable between baseline and follow-up. We adjusted for sociodemographic variables, health status, and baseline value of the outcome variable in the analyses.

To assess if intervention effects varied by sex, age, and income [25], we performed ANCOVA that included product terms of the group and sex, age, and income. We also conducted analyses stratified by sex, age, and income. In addition, we examined differences in intervention effects between those who perceived the intervention program and those who did not.

The missing rate in each analysis model ranged from 0% to 6.8%. For missing information, we used the R package mice to perform multiple imputations by chained equations, assuming missing at random [26]. Fifty data sets were created, and the combined results of each data set to obtain the estimates.

All analyses were conducted using R 3.6.3 (R Foundation for Statistical Computing, Vienna, Austria).

## Results

Baseline characteristics of the study participants are shown in Table 1. In the control group, the mean (SD) age was 57.3 (14.9) years, and 39.7% were male. In the intervention group, the mean (SD) age was 57.7 (14.8) years, and 40.6% were male. The prevalence of higher educational attainment was significantly greater in the intervention group than in the control group (6.0%) (Table 1).

**Table 1:** Baseline characteristics (*N* = 2518)

	Control *N* = 1727^a^	Intervention *N* = 791^a^	*P*-value^*^
Age	57.3 (14.9)	57.7 (14.8)	0.480
Age group			0.640
Young adults (25–49 years)	32.7%	30.8%	
Mid-aged adults (50–64 years)	30.1%	31.4%	
Older adults (65–84 years)	37.3%	37.8%	
Sex			0.715
Male	39.7%	40.6%	
Female	60.3%	59.4%	
Years of education			0.005
< 13 years	60.9%	66.8%	
≥ 13 years	39.1%	33.2%	
Annual household income			0.090
Do not know	6.0%	6.5%	
< 1 million yen	3.1%	3.6%	
1–2 million yen	9.1%	13.2%	
2–3 million yen	16.1%	14.4%	
3–5 million yen	22.7%	20.9%	
5–7 million yen	17.2%	17.9%	
7–10 million yen	15.9%	14.8%	
≥ 10 million yen	10.0%	8.8%	
Employment status			0.498
Worker	61.8%	60.3%	
Non-worker	38.2%	39.7%	
Marital status			0.490
Married	65.2%	63.7%	
Widowed/divorced/single	34.8%	36.3%	
Living status			0.117
Living alone	81.3%	78.6%	
Living with others	18.7%	21.4%	
Mental health	14.8 (5.5)	14.7 (5.6)	0.652
Self-rated health			0.227
Good	85.6%	83.6%	
Poor	14.4%	16.4%	

^a^Mean (SD); %

^*^Two sample t-test; Chi-squared test

The results of the comparison between those who were lost to follow-up and those included in the primary analyses are shown in Supplementary Table 1. Those lost to follow-up were more likely to be younger, male, and have lower educational attainment, poor self-rated health, and lower social trust (Supplementary Table 1).

Participants’ awareness of the intervention is shown in Table 3. In total, 19.3% of participants knew the project title, with females and those with a high income having higher awareness than males or those with a low income. The proportion of those who had seen the logo was 25.9%, and more females, younger adults, and those with a high income had seen the logo than their males, older, and low-income counterparts. The prevalence of those with campaign goods was 4.6%, with more females and younger adults having goods than males and middle-aged to older adults. The percentage of those who knew about the intergenerational contact events was 26.0%, and 2.9% of participants had participated. There were differences in event participation by sex and age (Table 2).

**Table 2:** Awareness of the project by sex, age, and income (*N* = 791)

	Those who knew the project title		Those who had seen the logo		Those who had goods		Those who knew about the ICEs		Those who had attended ICEs	
	%	*P*-value^1^	%	*P*-value^1^	%	*P*-value^1^	%	*P*-value^1^	%	*P*-value^1^
Total										
	19.2		25.8		4.6		26.0		2.9	
Sex										
Male	12.5	< 0.001	18.1	< 0.001	1.3	< 0.001	19.0	0.053	0.9	0.012
Female	23.8		31.1		6.8		30.9		4.3	
Age group										
Young adult	23.3	0.149	42.9	< 0.001	10.3	< 0.001	21.3	0.053	1.6	< 0.001
Mid-aged adult	17.9		22.1		1.3		25.4		0.4	
Older adult	17.0		14.9		2.6		30.4		6.0	
Household income										
Low income	16.5	0.029	19.2	< 0.001	4.2	0.663	26.3	0.901	3.7	0.184
High income	23.1		35.2		5.1		25.6		1.7	

*ICEs* intergenerational contact events

^1^Chi-squared test

The changes in outcome variables during the intervention period are shown in Table 3. The results of ANCOVA, which examined the effects of the intervention on social capital, are shown in Table 4. Social trust slightly increased from 3.48 to 3.49 in the intervention group. In contrast, it decreased from 3.46 to 3.42 in the control group. The group difference was significant, and the intervention group showed a positive change compared with the control group (coefficient = 0.065; 95% CI: 0.006, 0.125). Norm of reciprocity increased from 3.23 to 3.30 in the intervention group, but slightly decreased from 3.24 to 3.22 in the control group; the intervention group showed more significant improvement than the control group (coefficient = 0.084; 95% CI: 0.020, 0.149) (Tables 3 and 4).

**Table 3:** Social capital at pre- and post-intervention in the control and intervention groups (*N* = 2518)

	Control *N* = 1727						Intervention *N* = 791					
	Baseline			Follow-up			Baseline			Follow-up		
	Mean	95% CI		Mean	95% CI		Mean	95% CI		Mean	95% CI	
Social capital indicators												
Social trust	3.46	(3.42,	3.50)	3.42	(3.38,	3.46)	3.48	(3.42,	3.54)	3.49	(3.43,	3.55)
Norm of reciprocity	3.24	(3.19,	3.28)	3.22	(3.17,	3.30)	3.23	(3.17,	3.30)	3.30	(3.24,	3.36)
Emotional social support	4.61	(4.52,	4.69)	4.70	(4.62,	4.79)	4.57	(4.45,	4.70)	4.77	(4.64,	4.90)
Instrumental social support	4.79	(4.70,	4.88)	4.83	(4.74,	4.91)	4.70	(4.58,	4.83)	4.80	(4.68,	4.93)

*CI* confidence interval

**Table 4:** Effects of the community-based intervention on social capital (*N* = 2518)

	β	95% CI	
Social capital indicators			
Social trust	0.067	(0.007,	0.127)
Norm of reciprocity	0.085	(0.020,	0.150)
Emotional social support	0.094	(− 0.031,	0.220)
Instrumental social support	0.041	(− 0.091,	0.172)

Analysis of covariates. The dependent variable was the change in each social capital indicator. The independent variable was the allocated group (control, intervention). Covariates were sex, age, years of education, household income, marital status, living arrangement, employment status, mental health, self-rated health, and the baseline score for the outcome. Coefficients higher than 0 indicated a more significant improvement in the intervention group than the control group

*Β* coefficient, *CI* confidence interval

**Fig. 2:**
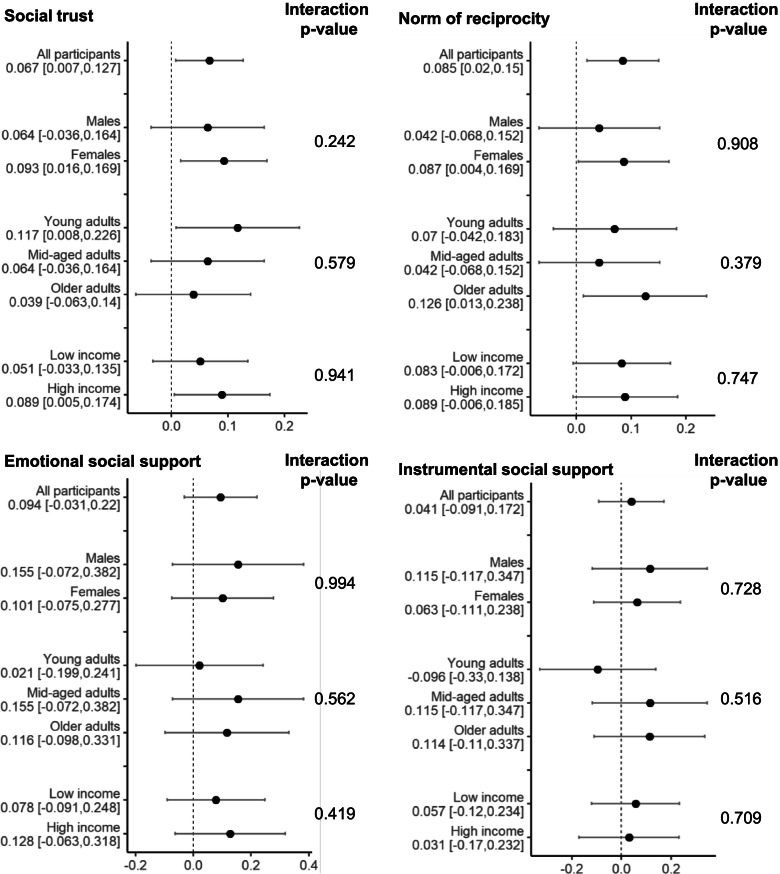
Effects of the community-based intergenerational intervention on social capital by sex, age, and income. Analysis of covariates adjusted for sex, age, educational attainment, household income, marital status, living arrangement, employment status, mental health, and self-rated health. Changing higher than 0 in each outcome indicated a greater improvement in the intervention group than the control group.

The results of the analyses that assessed the effect modification by sex, age, and household income, are shown in Fig. 2. No product term was significant. However, the stratified analyses demonstrated significant intervention effects on social trust among females, young adults, and high-income groups. In addition, significant intervention effects on norm of reciprocity were observed in females and older adults, but not in other groups (Fig. 2).

The differences in intervention effects on social capital by awareness of the intervention are shown in Supplementary Table 2. Norm of reciprocity was significantly improved in those who perceived the program compared with those that did not. Although the point estimate of social trust was higher than 0, it did not reach statistical significance (Supplementary Table 2).

## Discussion

We conducted a community-based intervention to increase intergenerational contact and examined its effects on social capital among adults living in an urban area. We found that social trust and norm of reciprocity were maintained and improved in the intervention group compared with the control group. Our findings suggested that this community-based intervention may contribute to enhancing community-level social capital among community-dwelling adults.

More females, young adults, and high-income participants knew the title/slogan/logo and had intergenerational greeting campaign goods compared with males, older adults, and low-income participants. This may be because those populations were more likely to have opportunities to learn about the intergenerational greeting campaign. We collaborated with local elementary and junior high schools, and these students participated in the campaign logo and slogan competition. The selected logo and slogan were printed on campaign goods and distributed to all students and their parents. These actions might have been effective in drawing the attention of schoolchildren’s parents to the campaign. In addition, campaign banners were displayed at the school gates and the train station. This might have meant that commuter workers, who were expected to be in the high-income group, remembered the campaign logo and slogan because they often saw them printed on the banner. To increase the prevalence of males who recognize intergenerational greeting campaigns, providing information and goods via organizations to which males are more likely to belong (e.g., community sports groups, fitness clubs) may efficiently raise awareness of the campaign among men.

More females and older adults participated in the intergenerational contact events compared with males and younger adults. It might reflect that the advertising tools we employed affected the behavior of these populations. For example, we provided information about the intergenerational contact event schedule and locations using community bulletin boards and placing fliers in community centers. Additionally, committee members and volunteer staff solicited their neighbors to visit the events. These tools tended to reach individuals who spend more time in the residential neighborhood, such as females and older adults. Thus, they were more likely to participate in the events. Another possible reason is the event dates. The intergenerational contact events were only held on weekdays, not weekends; therefore, employed people could not participate. Conducting the events on weekends and using other advertising tools may be effective in bringing together a broader population (i.e., young to middle-aged males).

Social trust was positively changed in the intervention group compared with the control group. Although there was no significant effect modification by age, sex, and income, the stratified analyses showed that significant differences were only found in females, young adults, and high-income participants. These were the same populations with a higher prevalence of knowing about the intergenerational greeting campaign slogan and logo or having campaign goods. It might indicate that the intergenerational greeting campaign could improve social trust. A previous study suggested that experiences of being treated well by others increased people’s sense of social trust [27]. Thus, increasing the frequency of greetings may have been perceived by neighbors as being treated well by others; the intergenerational greeting might enhance residents’ social trust. A possible reason for the non-significant effect among the older population was that the prevalence of those who regularly communicated with other generations was higher in older adults (40.1%) than in young adults (22.1%) [12]. Therefore, the effects of the greeting campaign, which aimed to promote intergenerational contact, on social trust might have been limited in the older group.

Norm of reciprocity was improved in the intervention group. There was no significant effect modification by sex, age, and income, but stratified analyses showed significant differences between the intervention and control groups in females and older adults. The prevalence of those who participated in intergenerational contact events was also higher in these populations. Participants shared a recreational activity with neighbors from other generations during the events and fostered their friendship. It resulted in building relationships in which they helped each other. For example, older participants played with participating children meaning their mothers could feel relaxed; young participants taught older adults how to use smartphones and social media (i.e., LINE). This mutual help during the events may have improved the norm of reciprocity among participants. In addition, as we expected, the influence of the intergenerational contact events expanded beyond the actual number of participants. However, the prevalence of event participants was low, even in the high participation groups (females: 4.3%, older adults: 6.0%), meaning the effect may be limited. The effect can increase if the number of event participants grows.

Inconsistent with our hypothesis, we did not find evidence of an intervention effect on emotional and instrumental social support. This may reflect that the intervention contents were inadequate to enhance community-level mutual help. A previous study suggested that factors needed to build mutual help relationships among residents were: 1) people requiring daily life support; 2) people having regular contact with neighbors; 3) people knowing their neighbors’ needs for daily life support; and 4) public organization (e.g., a community general support center) promoting mutual support in the area [28]. Our project committee included public organizations and promoted intergenerational interaction among residents. In addition, the intervention aimed to increase the frequency of intergenerational contact. Thus, we achieved factors 2 and 4, but the intervention may not fulfill factors 1 and 3. This may explain why we did not detect a significant increase in the frequency of exchanging social support. Another possible reason for this finding was that the total number of participants in the intergenerational contact events was small. The amount of the intervention might be insufficient to increase social support exchange. Further studies that have an extended follow-up period are needed to evaluate the effects of the intervention on social support.

Although several intervention studies promoting social capital have assessed its health effects [29], we did not examine the health effect of the intervention because enhancing community-level health status would have needed more time. Social trust and norm of reciprocity, which were significantly higher in the intervention group, are associated with a decreased risk for depression [22, 30]. Hence, this intervention may positively affect community-level mental health, but further studies are needed to clarify this effect.

The strength of this study was that it was a community-based intervention. Our findings would be beneficial for developing a strategy to improve community-level social capital. However, this study has several limitations. First, the response and follow-up rates were low. Although we used a random sampling method, selection bias might have occurred. We confirmed that there were significant differences in several variables between those lost to follow-up and those included in the primary analyses. Therefore, participants in this study may differ from the general population. Second, there might have been residual confounding, such as characteristics of the district. Although we adjusted for sociodemographic variables, conditions other than the intervention might not have been the same between the intervention and control groups.

## Conclusions

Our findings suggest that the community-based intervention positively affected social capital through social trust and norm of reciprocity among adults living in an urban area. This intervention may contribute to improving or sustaining community-level social capital.

## Supplementary Information

**Additional file 1: Supplementary Table 1.** Differences in baseline characteristics between those lost to follow-up and the sample for analysis (*N* = 5207)^1^. **Supplementary Table 2.** Differences in intervention effects in the intervention group between those who perceived the intervention program and those who did not (*N* = 791). **Supplementary Figure 1.** Intergenerational greeting campaign logo and slogan. **Supplementary Figure 2.** Intergenerational greeting campaign goods (banners, badges, and stationery).

## Data Availability

The datasets used during the current study are available from the corresponding author on reasonable request.
