# Supplementary material for: Effects of intergenerational contact on social capital in community-dwelling adults aged 25–84 years: a non-randomized community-based intervention
Source: BMC Public Health. 2022 Sep 24;22:1815. doi: 10.1186/s12889-022-14205-6 (PMC9508708; doi:10.1186/s12889-022-14205-6)
Supplement: Supplementary file 1 — Additional file 1: Supplementary Table 1. Differences in baseline characteristics between those lost to follow-up and the sample for analysis (N = 5207)1. Supplementary Table 2. Differences in intervention effects in the intervention group between those who perceived the intervention program and those who did not (N = 791). Supplementary Figure 1. Intergenerational greeting campaign logo and slogan. Supplementary Figure 2. Intergenerational greeting campaign goods (banners, badges, and stationery). [file 12889_2022_14205_MOESM1_ESM.pdf]

Supplementary Table 1. Differences in baseline characteristics between those lost to follow-up and the sample for analysis (N = 5207)[1]

Variables	PR	95% CI	
Group (control group)	0.995	(0.917,	1.080)
Age[2]	0.992	(0.989,	0.995)
Sex (males)	0.913	(0.840,	0.992)
Years of education (<13 years)	0.902	(0.826,	0.985)
Annual household income (<5 million yen)	0.958	(0.881,	1.041)
Employment status (Workers)	1.081	(0.981,	1.192)
Marital status (Married)	1.091	(0.989,	1.205)
Living status (Living alone)	0.984	(0.877,	1.105)
Mental health[2]	0.995	(0.987,	1.002)
Self-rated health (Good)	1.127	(1.016,	1.251)
Social trust[2]	0.930	(0.880,	0.984)
Norm of reciprocity[2]	0.995	(0.943,	1.049)
Emotional social support[2]	0.991	(0.954,	1.029)
Instrumental social support[2]	0.996	(0.960,	1.033)

PR, prevalence ratio; CI, confidence interval

The reference category was shown in parentheses.

[1] Poisson regression model. The dependent variable was refusing to participate in the follow-up survey. PR higher than 1 indicates that higher prevalence of loss to follow-up.

[2] The variables were included as a continuous variable.

Supplementary Table 2. Differences in intervention effects in the intervention group between those who perceived the intervention program and those who did not (N = 791)

	β	95% CI
Social capital indicators		
Social trust	0.075	(−0.026, 0.176)
Norm of reciprocity	0.124	(0.011, 0.236)
Emotional social support	-0.055	(−0.511, 0.400)
Instrumental social support	0.287	(−0.182, 0.755)

β; coefficient, CI; confidence interval

Analysis of covariates. The dependent variable was the change in each social capital indicator. The independent variable was awareness of the intervention (those who perceived the intervention or not). Covariates were sex, age, years of education, household income, marital status, living arrangement, employment status, mental health, self-rated health, and the baseline score for the outcome. Coefficients higher than 0 indicated a more significant improvement in the intervention group than the control group.

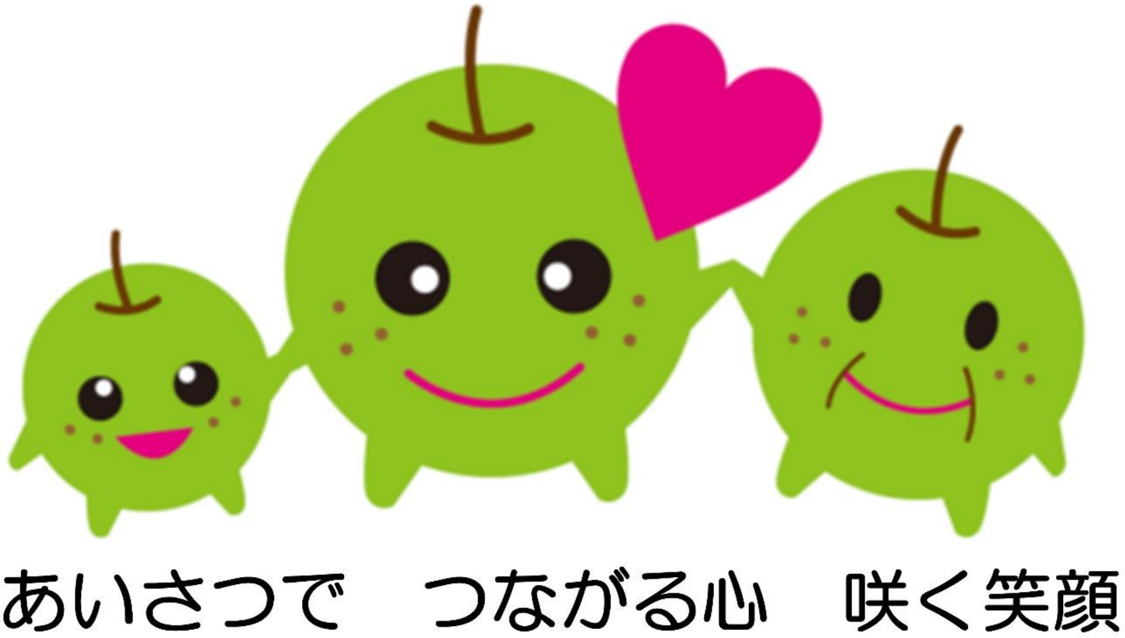

Supplementary Figure 1. Intergenerational greeting campaign logo and slogan

The slogan means “Greetings make people smile and foster relationships.”

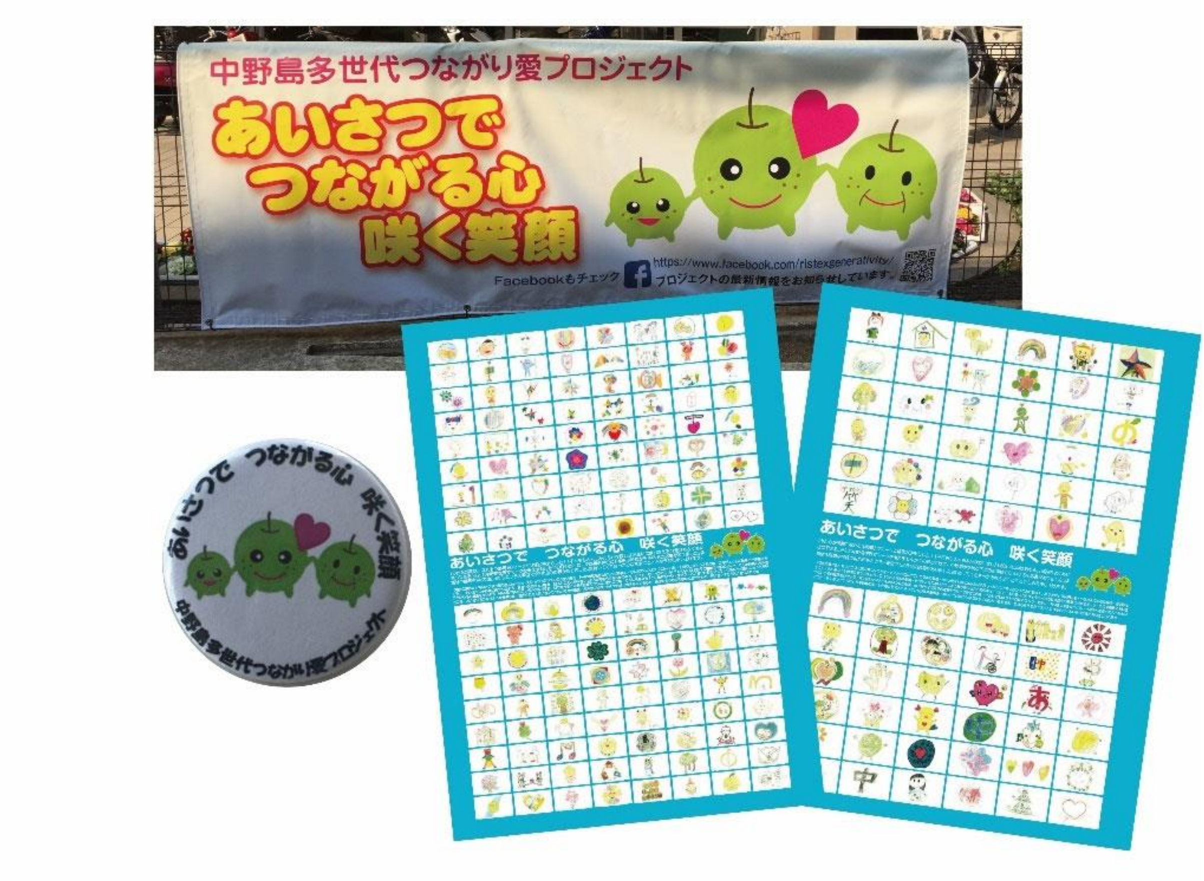

Supplementary Figure 2. Intergenerational greeting campaign goods (banners, badges, and stationery)

The banners were hanging at the school gate and the train station. The badges and stationery were distributed to elementary and junior high school children and their parents.
